# Characterization of an Endophytic Antagonistic Bacterial Strain *Bacillus halotolerans* LBG-1-13 with Multiple Plant Growth-Promoting Traits, Stress Tolerance, and Its Effects on Lily Growth

**DOI:** 10.1155/2022/5960004

**Published:** 2022-08-25

**Authors:** Jun-lian Gao, Mohammad Sayyar Khan, Yu-chen Sun, Jing Xue, Yunpeng Du, Chunhong Yang, Vladimir K. Chebotar, Vladimir S. Tikunov, Ivan N. Rubanov, Xuqing Chen, Xiuhai Zhang

**Affiliations:** ^1^Institute of Grassland, Flowers and Ecology, Beijing Academy of Agriculture and Forestry Sciences, Beijing 100097, China; ^2^Microbiology Division, Institute of Biotechnology and Genetic Engineering (IBGE), The University of Agriculture, Peshawar 25000, Pakistan; ^3^Institute of Botany, Chinese Academy of Sciences, Beijing-100093, China; ^4^All-Russia Research Institute for Agricultural Microbiology, St. Petersburg, Pushkin-8, 196608, Russia; ^5^Lomonosov Moscow State University, Department of Geography, 119991 Moscow, Russia

## Abstract

Microbial inoculants are an important tool for increasing arable land productivity and decreasing mineral fertilizer application. This study was aimed at isolating and identifying endophytic antagonistic bacteria from lily (*Lilium davidii* var. *unicolor*) roots grown in Northwestern China and at evaluating their antifungal activity and plant growth-promoting characteristics. For this purpose, endophytic bacteria were isolated from plant roots, and plant growth-promoting strains were identified. One bacterial strain, isolated from the root part, was identified as *Bacillus halotolerans* based on 16S rRNA gene sequence analysis and was designated as LBG-1-13. The strain showed antagonistic activities against important plant pathogens of lily including *Botrytis cinerea*, *Botryosphaeria dothidea*, and *Fusarium oxysporum*. The highest percentage of growth inhibition, i.e., 71.65 ± 2.39%, was observed for LBG-1-13 against *Botryosphaeria dothidea* followed by 68.33 ± 4.70% and 48.22 ± 4.11% against *Botrytis cinerea* and *Fusarium oxysporum*, respectively. Meanwhile, the isolated strain also showed plant growth-promoting traits such as the production of indole-3-acetic acid (IAA), siderophore, ACC deaminase, and phosphate solubilization activity. The strain showed ACC deaminase activity and was able to cleave 58.41 ± 2.62 nmol *α*-ketobutyrate (mg protein)^−1^ min^−1^. The strain exhibited tolerance to salt and drought stress in an *in vitro* experiment. The strain LBG-1-13 was able to grow in the presence of 10% NaCl and 20% polyethylene glycol (PEG) in the growth medium. Inoculation of *Lilium* varieties, Tresor and Bright Diamond, with LBG-1-13 enhanced plant growth under greenhouse and field conditions, respectively. All these results demonstrated that *Bacillus halotolerans* LBG-1-13 could be utilized as a good candidate in the biocontrol of lily disease and plant growth promotion in sustainable agriculture.

## 1. Introduction

The world population is growing at an alarming rate and is expected to reach 9.2 billion in 2050. This means that global agricultural production has to be increased by 60-70% from the current levels to keep pace with the growing food demand in the next 30 years [1]. Enhanced agricultural production can be achieved through stimulating the yield potential of crop plants as well as protecting them from phytopathogens. Commercial fertilizers and pesticides have been extensively used to maximize agricultural production in the past. However, due to the growing concerns regarding the adverse effects of synthetic agrochemicals on human and environmental health, more environment-friendly alternatives are encouraged to be utilized by farmers [2]. In addition, the excessive use of synthetic agrochemicals may trigger the development of resistance in the phytopathogens that further necessitates the need for alternative approaches [3].

The utilization of biofertilizers and biopesticides has become a preferred alternative of synthetic-based chemical products for pest biocontrol and plant growth improvement. The market turnover of these products exceeded 4 billion US dollars and has been growing by 5-10% per year [4]. Bioinoculants are especially important for China where freshwaters are strongly polluted with nitrogen and phosphorous leached from hilly arable lands with intensive agriculture systems and where eutrophication is an urgent problem [5]. Endophytic microorganisms dwell inside various plant tissues and play an important role in the overall plant development and adaptation to biotic and abiotic stresses [6]. Endophytes use the endosphere of the plant as a unique ecological niche that protects them from environmental changes. This association between the endophytes and their host plants has been established as a result of millions of years of evolution [7, 8]. Endophytic bacteria colonize the same ecological niche as pathogens and work as biocontrol agents against plant pathogens [9–11].

Endophytic bacteria and plant growth-promoting rhizobacteria (PGPR) have direct and indirect beneficial effects on plants through different underlying mechanisms [12]. They assist the associated plants in mineral acquisition, fight against phytopathogens, and confer tolerance against drought and salt stresses [13, 14]. The key mechanisms lying at the core of PGPR-mediated plant growth-promoting effects and disease resistance in the associated plants include the synthesis of plant growth hormones, ACC deaminase, organic acids, siderophore, nitrogen fixation, and phosphate solubilization [15]. There have been several reports that described the isolation and characterization of endophytic bacteria and PGPR with the potential of plant growth-promoting and disease resistance traits. These beneficial effects are mainly due to the production of enzymes and hormones directly involved in plant growth promotion and the synthesis of antimicrobial secondary metabolites and defense-related compounds [16, 17]. The microbial-based pathogen resistance is conferred through various mechanisms such as the secretion of antimicrobial compounds that degrade cell walls of pathogens and block the rhizosphere colonization of pathogens [18]. Besides plant growth-promoting and antimicrobial potential, the role of plant-associated endophytic bacteria in plant abiotic stress tolerance has been well documented [19–21]. Several endophytic bacteria isolated from diverse hosts conferred salt and drought tolerance to inoculated plants [22–24].

In the recent past, a large number of *Bacillus* species have been characterized as important producers of plant growth-promoting substances, secondary metabolites, and bioactive compounds [25, 26]. The endospore-forming potential of *Bacillus* species is also very important for agriculture development as these have long shelf lives and are resistant to desiccation and heat exposure. Moreover, they have negligible or no side effects on the diversity and compositions of root-associated plant microbial communities [27, 28]. On the contrary, a recent study conducted on the impact of coinoculation of arbuscular mycorrhizal fungi and *Bacillus megaterium* revealed changes in bacterial diversity in the rhizosphere of turmeric [29]. However, changes in the microbial activities and diversity were positive in coinoculated treatments as compared to treatments with a sole application of AM and *B. megaterium*. Some endophytic isolates of the genus *Bacillus* including *Bacillus amyloliquefaciens*, *Bacillus subtilis*, and *Bacillus licheniformis* have been evaluated for their positive impact on plant growth and fungal disease resistance [30–32]. Due to the immense potential of *Bacillus* species as agents of plant growth promotion and defense against phytopathogens, several *Bacillus*-based products are used as bioinoculants and biocontrol agents in sustainable agriculture [33]. In addition to the use of existing PGPR and endophytes, research should be focused on the utilization of diverse sources of soil and plant hosts for new strains with multiple beneficial effects on plant development and disease resistance. Medicinal plants are known to harbor potential endophytic microbes, due to their bioactive compounds, and these microbes are responsible for the host's pharmaceutical properties [34, 35]. Therefore, medicinal plants should be investigated for isolation and characterization of more effective PGPR and endophytes.

Lilies (genus *Lilium* L.) belong to a group of perennial, herbaceous, bulbiferous, and large flowering plants growing from bulbs. The *Lilium* species are cultivated as ornamental plants throughout the world as well as used as important edible plants and biological medicinal products [36]. They are important both as garden plants and as pot cultured and cut flowers [37]. Several studies reported that the agrochemical characteristics of soil were changed and the microbial functional diversity was suppressed in soils under continuous cropping of lily [37, 38]. Microbial composition and functional diversity were changed in the rhizosphere of *L. davidii* under continuous monoculture [39]. Recent research revealed that plants can shape their rhizosphere microbiome and affect the diversity and structure of the microorganism community in the rhizosphere [40].

During a survey on endophytic antagonistic bacteria against pathogenic fungi of lily, we found that a strain, designated strain LBG-1-13 isolated from the root of lily, had obvious antagonistic activities against a variety of lily pathogenic fungi. Considering its potential value in the biological control of lily diseases, we further studied this strain in the present study. The purposes of the present study were as follows: (i) to isolate and identify endophytic antagonistic bacteria from the roots of *Lilium davidii* var. *unicolor*, (ii) to determine the characters of plant growth-promoting and tolerances to salt and drought of this strain, and (iii) to investigate its effects on growth of lilies under greenhouse and field conditions.

## 2. Materials and Methods

### 2.1. Isolation of Endophytic Bacteria from Lily Roots and Their Antagonistic Activity Against Pathogenic Fungi of Lily

In summer of 2016, three seedlings of lily (*L. davidii* var. *unicolor*) were collected in the District of Qilihe (35°50′8.38^″^N, 103°53′55.40^″^E) of Lanzhou City in Gansu Province, China. The roots were cut off and mixed. Then, endophytic bacteria were isolated from the surface-sterilized roots of lily plants. Root sterilization was done with 70% ethanol for 1 min and 2% sodium hypochlorite for 10 min, followed by rinsing in a sterile distilled water for 10 min. For evaluation of the efficacy of the surface sterilization, the samples were inoculated on tryptic soy agar (TSA) (BD Diagnostics, Sparks, Maryland, USA) triplicate plates. The surface contamination-free samples were cut into smaller pieces (~1 cm) and inoculated on LB agar [41]. After aerobic incubation for 3 days at 30°C, single colonies were picked up and tested their antagonistic activity against pathogenic fungi of lily, using the method described previously [42]. The pathogenic fungi included *Botryosphaeria dothidea* (ACCC 37263), which causes leaf dieback/lily leaf tip blight disease in lily plants, *Botrytis cinerea* (ACCC 36423), which causes gray mold disease on lily, and *Fusarium oxysporum* (ACCC 37279), which causes root and bulb rot disease on lily. These fungal strains were provided by the Agricultural Culture Collection of China.

### 2.2. Molecular Identification of Endophytic Bacterial Isolate

Molecular identification of the endophytic bacterial isolates was done with amplification and sequencing of 16S rRNA gene. Bacterial genomic DNA was extracted from bacterial colonies. A bacterial colony was scraped off the LB medium plate and was suspended in 100 *μ*l ddH_2_O and lysed for 10 min boiling and subsequent freezing for 5 min. The sample was centrifuged at 13,000 rpm for 2 min, and the supernatant was used as the PCR template. Universal 27F and 1492R were used for 16S rRNA gene amplification [43]. The 25 *μ*l PCR reaction contained 1 *μ*l (0.5–10.0 ng) template DNA, 12.5 *μ*l of Premix Taq Version 2.0 (TaKaRa Bio Group), 0.5 *μ*l (10 *μ*M) of primers 27F and 1492R each, and 10.5 *μ*l of ddH_2_O. The thermocycler conditions were set as initial denaturation at 94°C for 3 min, 30 cycles of denaturation at 94°C for 40 s, annealing at 55°C for 1 min, extension at 72°C for 2 min, and a final overall extension at 72°C for 10 min. Purification of PCR product was done with the QIAquick PCR Purification Kit (Qiagen, Hilden, Germany). The purified PCR product was then sequenced through the ABI 3730 sequencer following the manufacturer's protocols through Beijing Biomed Gene Technology Co. Ltd. The endophytic bacterial sequences of the 16S rRNA gene were then compared with the available homologous sequences in the EzBioCloud database (http://www.ezbiocloud.net/) for phylogenetic analysis [44].

### 2.3. Detection of Indole Acetic Acid (IAA)

Quantitative detection of indole acetic acid (IAA) was done according to the method of Libbert and Risch [45]. Briefly, the bacterial strain was cultivated in fresh King's B medium (peptone/tryptone 20 g, K_2_HPO_4_ 1.15 g, MgSO_4_·7H_2_O 1.5 g, l-tryptophan 0.5 g, glycerol 15 ml, and distilled water 1000 ml; pH 6.8~7.0) at 30°C for 24~48 hr. Then, 2 ml bacterial culture was mixed with 2 ml of Salkowski's reagent (0.5 M FeCl_3_ and 35% HClO_4_, 2 : 100) in a glass tube, and the mixture was left in the dark for 30 min at room temperature. A noninoculated medium was used as a negative control, and the IAA solution (100 mg/L) was used as a positive control. The formation of pink color in the tube indicated indole production by the organism.

### 2.4. Phosphate Solubilization Assay

Phosphate solubilization of the endophytic bacterial strain was conducted qualitatively as previously described by Mehta and Nautiyal [46]. The endophytic strain was cultivated in Pikovskaya medium (1% glucose, 0.05% (NH_4_)_2_SO_4_, 0.01% MgSO_4_·7H_2_O, 0.02% KCl, 0.02% NaCl, 0.05% yeast extract, 0.0002% FeSO_4_·7H_2_O, 0.0002% MnSO_4_·H_2_O, and 1.5% agar 1.5). The strain was evaluated for its ability to grow on Pikovskaya medium using either tricalcium phosphate (Ca_3_(PO_4_)_2_) or Aluminium phosphate (Al_3_PO_4_) as the sole source of phosphate. Halos around the colonies indicating solubilization of inorganic phosphate were observed after 7 days of cultivation.

### 2.5. Siderophore Detection

Siderophore production in the endophytic strain was determined on chrome azurol S (CAS) medium according to the previously described method [47]. For the preparation of 1 l blue agar, 60.5 mg CAS was dissolved in 50 ml water and mixed with 10 ml iron (III) solution (0.001 mol l^−1^ FeCl_3_·6H_2_O, and 0.01 mol l^−1^ HCl). This solution was slowly added to 72.9 mg CTAB dissolved in 40 ml water, and the resultant dark blue solution was autoclaved. A mixture of 780 ml H_2_O, 100 ml 10 times MM9 salts (3 g l^−1^ KH_2_PO_4_, 10 g l^−1^ NH_4_Cl, and 5 g l^−1^ NaCl), 30.24 g Pipes, and 15 g agar was prepared with pH 6.8 adjusted with NaOH and was autoclaved. Upon cooling to 50°C, 10 ml glucose (20% *w*/*v*), 1 ml CaCl_2_ (100 mol l^−1^), 1 ml MgSO_4_ (1 mol l^−1^), 4 ml thiamine (500 mg ml^−1^), 4 ml nicotinic acid (500 mg ml^−1^), and 100 ml of the above mentioned dark-blue solution were added. The overnight bacterial culture was spotted on the CAS medium thus obtained. Siderophore production was evidenced by the formation of orange to yellow halos around the colonies after 7 days of incubation at 30°C.

### 2.6. ACC Deaminase Activity Assay

The ability of the strain to show ACC deaminase activity was determined quantitatively through a colorimetric microplate assay as previously described by Penrose and Glick [48]. Total protein content in the sample was determined following Bradford [49], with a standard curve of bovine serum albumin ranging between 0 and 4 mg ml^−1^ by measuring the absorbance at 595 nm.

### 2.7. In Vitro Salt and Drought Tolerance Assay of LBG-1-13

Salt tolerance of the strain LBG-1-13 was determined through an *in vitro* test. Ten salt (NaCl) concentrations, i.e., from 1% to 10%, were used in the experiment. The overnight bacterial culture was inoculated to fresh LB medium supplemented with various salt concentrations. The samples were incubated at 28°C for 24 hr, 48 hr, and 72 hr. Bacterial growth was checked spectrophotometrically by measuring OD at 600 nm at the mentioned salt concentrations in the LB medium. Drought stress tolerance of the strain LBG-1-13 was assayed in LB medium supplemented with different concentrations, i.e., 0% to 20% of polyethylene glycol (PEG). Samples were kept at 28°C for one, two, and three days, after which the OD of each sample was measured at 600 nm.

### 2.8. Plant Growth Promotion in Pot Experiment

The effects of the strain LBG-1-13 were evaluated on Tresor, a commercially cultivated *Lilium* variety in China. A greenhouse experiment was conducted using the method described previously [42] with minor modification. The modification included the following: the strain LBG-1-13 was inoculated after sowing for 45 days. The optical density (OD) of the bacterial suspension was measured at 600 nm with a spectrophotometer and adjusted to an OD_600_ of 1 that corresponded to 10^9^ colony-forming units (CFUs). The culture was diluted 2 times with normal water and then added to *Lilium* seedlings, each pot added with 200 ml. After inoculation, the plants were further grown for 43 days; then the root system was washed from the pots and was analyzed using the same statistical analysis method just as described previously [45].

### 2.9. Plant Growth Promotion in Field Experiment

The isolated bacterial strain LBG-1-13 was further tested for plant growth promotion effects on the selected *Lilium* variety, Bright Diamond, a commercial variety cultivated in China. From May 2018 to August 2018, a field experiment was conducted in the Fangshan District of Beijing, China. The soil physicochemical characteristics of the field were as follows: pH value was 7.4, total nitrogen was 0.132%, fast-acting potassium was 145 mg/kg, available phosphorus was 71.9 mg/kg, EC value (Electrical Conductivity) was 576 *μ*S/cm, and organic matter was 20.1 g/kg. Each field plot had an area of 40 m^2^ (20 m × 2 m). More than 1000 lilies grew in a plot, with 8 rows of plants spaced 0.15 m apart. Three plots were employed for both the treatment and the control. Fertilizers at a concentration of 25 kg (NH_4_)_2_HPO_4_ + 40 kg (P_2_O_5_ + K_2_O) ha^−1^ were applied as a basal dose to all the plots before sowing. Before inoculation, the overnight culture of LBG-1-13 in 5 ml LB was further inoculated in 200 ml LB and was cultured for 24 hr at 30°C with 220 rpm shaking, then inoculated in 1000 ml LB, and was kept to grow for 2-3 days until the bacterial suspension reached a concentration of 2.2 × 10^9^ cfu ml^−1^ or OD_600_ about 3.0. The culture was then diluted 10 times with normal water, and the lily bulbs were soaked for half an hour in the diluted bacterial solution. The plots were arranged in a randomized block design with each treatment replicated three times. The plants were irrigated daily with tap water. After 3 months, the growth status of the lilies in the field was observed qualitatively.

### 2.10. Statistical Analysis

The collected data were analyzed through Analysis of Variance (ANOVA) using SAS 9.4 software and means were compared by Student's *t*-test and Tukey's Honestly Significant Difference (HSD) at (*p* ≤ 0.05).

## 3. Results

A total of 40 endophytic bacterial strains were isolated from the roots of *Lilium davidii* var. *unicolor*. After screening for the antagonistic activities against three kinds of common lily pathogenic fungi, an endophytic bacterial strain with multiple antagonistic activities designated strain LBG-1-13 was found.

**Figure 1:**
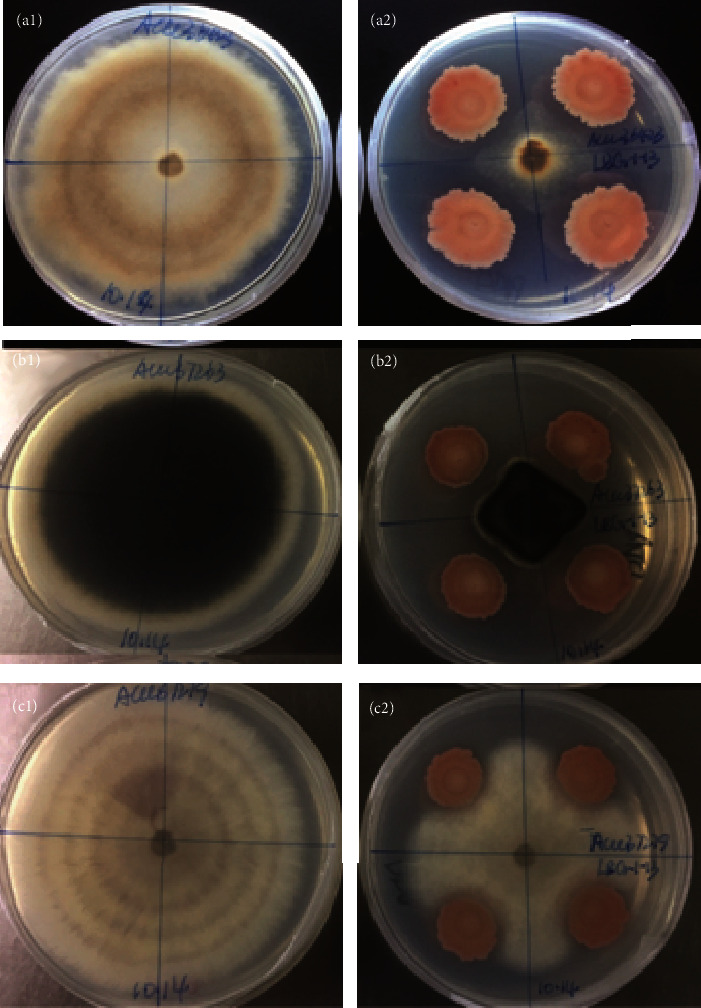
Antagonistic activity of the endophytic bacterial strain LBG-1-13 against three fungal pathogens. A fungus plug was inoculated into the center of the potato dextrose agar (PDA) medium surrounded by four spots of bacterial inoculum. Plates (A1), (B1), and (C1) are controls of *Botrytis cinerea* (ACCC 36423), *Botryosphaeria dothidea* (ACCC 37263), and *Fusarium oxysporum* (ACCC 37279), respectively. Plates (A2), (B2), and (C2) contain dual cultures of strain LBG-1-13 and the corresponding fungal pathogens.

The endophytic bacterial strain LBG-1-13 showed significantly high potential of mycelial growth inhibition of the test fungal pathogens, *B. cinerea* (ACCC 36423), *B. dothidea* (ACCC 37263), and *F. oxysporum* (ACCC 37279) (Figure 1). The growth inhibition of pathogenic fungi on PDA plates was measured as zones of inhibition in percentage values. The LBG-1-13 strain exhibited the highest percentage of growth inhibition, i.e., 71.65 ± 2.39% against *B. dothidea* followed by 68.33 ± 4.70% and 48.22 ± 4.11% against *B. cinerea* and *F. oxysporum*, respectively. These results suggested that the endophytic strain LBG-1-13 is highly effective against the tested disease-causing phytopathogens.

**Figure 2:**
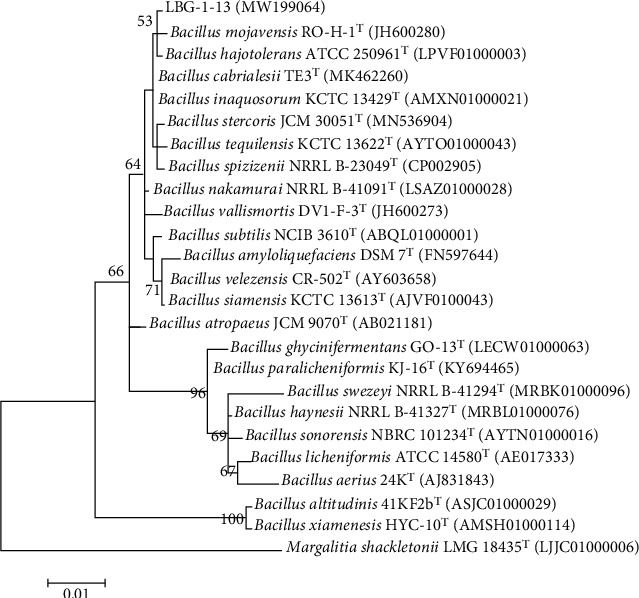
Phylogenetic analysis of 16S rRNA gene sequences of the bacterial endophyte LBG-1-13. Sequences were aligned through “Clustal W” using MEGA 7 software. Phylogenetic tree was constructed using Maximum Likelihood method. Bootstrap values are shown as percentages of 1000 replicates; values below 50% are not indicated. *Margalitia shackletonii LMG*18435 (LJJC01000006) was used as an outgroup.

The strain LBG-1-13 was further identified as a member of the genus *Bacillus* based on the 16S rRNA gene sequence analysis. A 1408 bp long 16S rRNA gene sequence was obtained and then submitted to GenBank under accession number MW199064. A total of 25 homologous 16S rRNA sequences including the query sequence of strain LBG-1-13 were aligned, and a phylogenetic tree was constructed (Figure 2). In the phylogenetic tree, the strain LBG-1-13 formed a separate group together with *B. halotolerans* ATCC 25096^T^ and *Bacillus mojavensis* RO-H-1^T^ and shared the highest sequence similarities (99.93%) with *B. halotolerans*.

**Figure 3:**
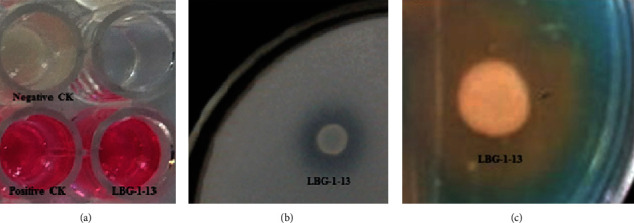
Qualitative detection of plant growth-promoting traits in the strain *Bacillus halotolerans* LBG-1-13. (a) Qualitative detection of IAA showing a change of coloration from yellow to pink. (b) Phosphate solubilization activity of strain LBG-1-13 was assayed on the Pikovskaya medium as a clearing area surrounding bacterial colonies. (c) Production of siderophore was confirmed by a change of color from blue (−) to yellow/orange (+) as indicated by chrome azurol S assay.

The endophytic strain LBG-1-13 was further evaluated for plant growth-promoting traits through several qualitative and quantitative tests which included the detection of indole acetic acid (IAA), siderophores, phosphate solubilization, and ACC deaminase. Production of IAA in the isolate was confirmed through a qualitative test showing a change of color of the culture supernatant from yellow to pink (Figure 3(a)). Phosphate solubilization potential of the isolated strain *B. halotolerans* LBG-1-13 was assayed on solid PVK medium supplemented with either Ca_3_(PO_4_)_2_ or Al_3_PO_4_ as the sole source of inorganic phosphate. The endophytic strain LBG-1-13 showed continued growth on both media for a longer incubation time and produced halos around the colonies indicating the ability of the strain to utilize inorganic phosphate in the medium (Figure 3(b)). Siderophore production in the isolate was assayed by a qualitative test that confirmed siderophore production through a change of color from blue to orange-yellow (Figure 3(c)). The potential of the isolated *B. halotolerans* LBG-1-13 to produce 1-aminocyclopropane-1-carboxylate (ACC) deaminase was assayed through a quantitative test. The strain showed ACC deaminase activity and was able to cleave 58.41 ± 2.62 nmol *α*-ketobutyrate (mg protein)^−1^ min^−1^. It has been reported that >20 nmol *α*-ketobutyrate (mg protein) ^−1^ min^−1^ is sufficient to initiate plant-growth-promoting effects [41].

**Figure 4:**
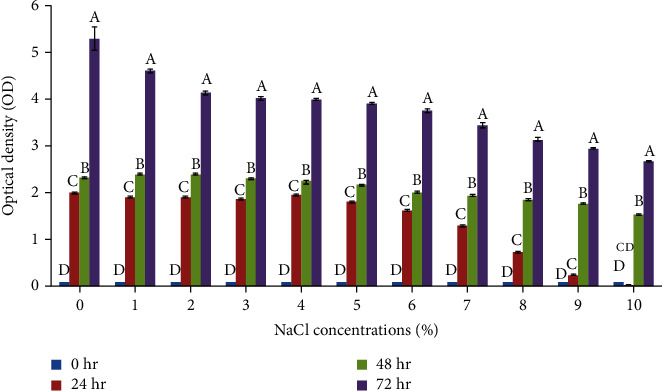
Impact of various salt (NaCl) concentrations on the growth of LBG-1-13 over different incubation times. Data are averages ± SD values (*n* = 3). Means having the different letters within each concentration differ significantly at (*p* ≤ 0.05) by Tukey (HSD).

**Figure 5:**
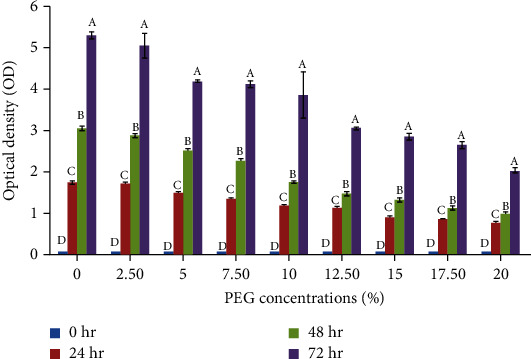
Effect of different polyethylene (PEG) concentrations on the growth of LBG-1-13 over different incubation times. Data are averages ± Standard Deviation (SD) values (*n* = 3). Means having the different letters within each concentration differ significantly at *p* ≤ 0.05 by Tukey (HSD).

Salt tolerance potential of LBG-1-13 was determined in LB medium supplemented with various NaCl concentrations. Salt stress negatively affected the bacterial growth after 24 hr of incubation in a medium with 1% NaCl (Figure 4). Further increase in salt concentration up to 6% NaCl had a nonsignificant effect on the growth of LBG-1-13. However, the effect of high salt concentrations was more pronounced from 7% to 10% NaCl concentrations. Drought stress tolerance of LBG-1-13 was tested by measuring growth in a medium supplemented with various PEG concentrations. Bacterial growth was checked after 24 hr, 48 hr, and 72 hr of incubation (Figure 5). PEG concentration of 2.5% in the medium had no significant effect on bacterial growth as compared to bacterial growth on 0% PEG. Further increase in PEG concentration reduced the bacterial growth and this trend continued till 10% PEG concentration. Further reduction in bacterial growth was observed in medium supplemented with 12.5% to 20% concentration. These results indicated the potential of LBG-1-13 to tolerate even a high concentration of PEG in the growth medium.

**Figure 6:**
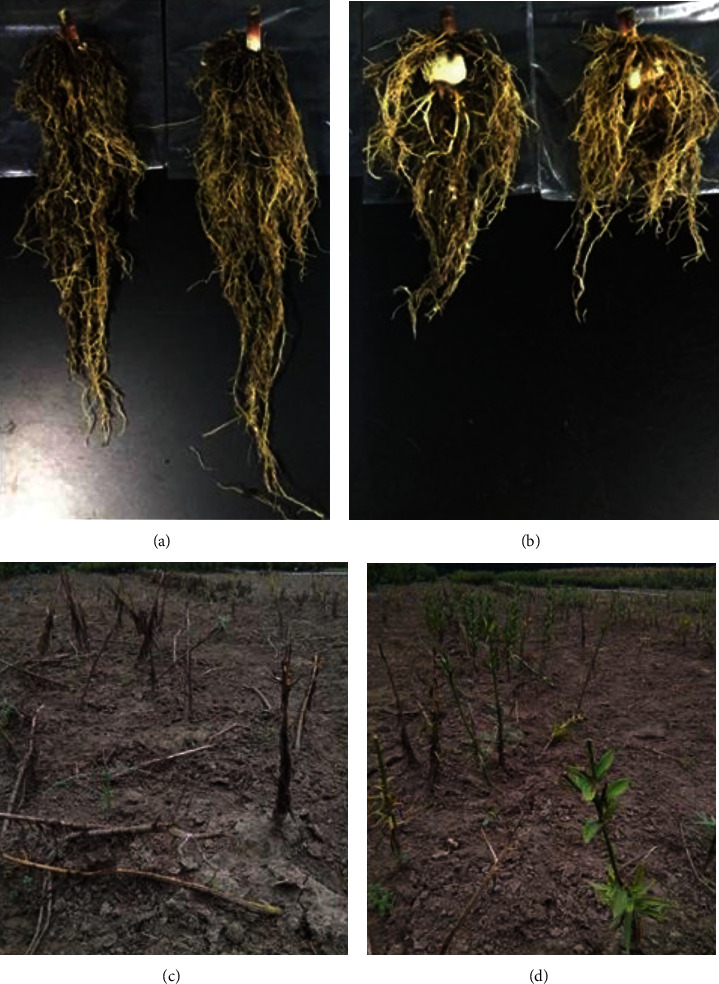
The effect of inoculation of endophytic bacteria LBG-1-13 on Tresor and Bright Dimond. Development of the root system of *Lilium* variety Tresor. (a) Inoculation with LBG-1-13; (b) control (inoculation with only LB medium). Extending the growth period of *Lilium* variety, Bright Diamond, by the strain LBG-1-13. After inoculation for more than 3 months, the inoculated lily plants still have much more green leaves (d) when compared to uninoculated control plants (c).

The effects of *B. halotolerans* LBG-1-13 inoculation on the Tresor lily variety were evaluated in the pot experiments under greenhouse conditions. Several growth parameters were checked between the inoculated and noninoculated control plants (Table 1). Among the tested growth parameters, plant height, leaf length, and leaf width were observed as nonsignificant between the inoculated and noninoculated plants. However, the inoculated plants showed significantly higher root length and root dry weight than those of the noninoculated control plants. The LBG-1-13-inoculated plants of the Tresor variety showed significantly higher (*p* < 0.05) root length than that of noninoculated control plants (Table 1 and Figures 6(a) and 6(b)). Root dry weight was also found to be significantly different between the inoculated and noninoculated control plants of the Tresor variety. Inoculated plants showed significantly higher (*p* < 0.05) root dry weight (11.66 ± 1.66 g) as compared to that of noninoculated control plants which showed 4.9 ± 0.70 g root dry weight. As a whole, the LBG-1-13-inoculated Tresor variety showed significant improvement in root growth as compared to that of the noninoculated control.

The effects of inoculation on the *Lilium* variety, Bright Diamond, in the field experiments were observed phenotypically. In general, the strain LBG-1-13 showed a tendency toward promoting lily growth and extending its growth period. In the entire field experiment, the inoculated lily grew significantly better than the noninoculated control plants during the seedling stage and the flowering period. After the lily flowering period (after inoculation of the strain for more than 3 months), all noninoculated control plants turned yellowish and died (Figure 6(c)), whereas most of the plants inoculated with strain LBG-1-13 were still green and had much more green leaves than the uninoculated controls (Figure 6(d)). These results suggest that the inoculated strain LBG-1-13 extended the growth period of lily plants.

## 4. Discussion

A new endophytic bacterial strain LBG-1-13 was isolated from the root of a *Lilium* variety, *L. davidii* var. *unicolor*, grown in Lanzhou, Gansu Province, Northwestern China. The strain was identified as *B. halotolerans* through molecular analysis of the 16S rRNA gene sequence. The strain showed antagonistic activity against some important fungal phytopathogens. Further tests confirmed that the isolated strain harbored plant growth-promoting effects and practically improved the root growth of the tested lily varieties.

In the present study, the *B. halotolerans* LBG-1-13 exhibited variable antagonistic potential against fungal phytopathogens, i.e., *B. dothidea*, *B. cinerea*, and *F. oxysporum*. These phytopathogens were previously reported as agents of serious diseases in several crop plants including species of *Lilium* [50]. The antimicrobial nature of *B. halotolerans* strain isolated from various hosts has been demonstrated in previous studies. Therefore, the antagonistic effects of the isolated strain LBG-1-13 against the phytopathogens causing diseases in the genus *Lilium* would be of high interest. Slama et al. [51] reported screening of *B. halotolerans* isolates, designated as BFOA1 and BFOA4, which were highly active against *F. oxysporum* f. sp. *albedinis*. The strains were also found to be very active against several *Fusarium* species. The isolated bacterial strains also showed strong activities against *Botrytis cinerea* and other major phytopathogens including *Phytophthora infestans*, *Alternaria alternata*, and *Rhizoctonia bataticola*. The study concluded that *B. halotolerans* could be used as an effective warden against fungal infection, particularly *Fusarium* infection in plants. Furthermore, a recent study demonstrated the biocontrol potential of *B. halotolerans* QTH8 against *Fusarium pseudograminearum*, the causal agent of wheat crown rot disease [52]. The isolated strain QTH8 also inhibited the mycelial growth of *Hainesia lythri*, *Pestalotiopsis* sp., *Botrytis cinerea*, *Curvularia lunata, Phyllosticta theaefolia*, *Fusarium graminearum*, *Phytophthora nicotianae*, and *Sclerotinia sclerotiorum*. This enhanced fungal resistance might be due to the production of plant defense-related compounds and bioactive secondary metabolites. Previous studies have revealed the production of secondary metabolites including bacteriocins, fengycin, surfactin, iturin, bacillibactin, and acetoin in *Bacillus* species [53–55].

Members of the genus *Bacillus* are well known for their potential of possessing plant growth-promoting characteristics. Several *Bacillus* species characterized from diverse hosts showed the potential of plant growth promotion through the production of ACC (deaminase), IAA, siderophore, and phosphate solubilization [56]. The isolated strain LBG-1-13 showed the production of IAA that directly stimulates plant growth. Previous studies reported indole production in the rhizospheric byendophytic *Bacillus* isolates [57, 58]. El-Akhdar et al. [59] reported the production of IAA in the strain *B. halotolerans* MSR-H4, isolated from the rhizospheric soil of wheat plants. The isolated strain LBG-1-13 in the present study showed a phosphate solubilization potential. Previous studies have demonstrated phosphate solubilization as one of the key mechanisms of *Bacillus* species to stimulate plant growth promotion under normal and stress conditions [60, 61]. The isolated strain LBG-1-13 showed siderophore production. In the microbe-plant association, siderophores play a crucial role as iron chelators and ensure the availability of traces of iron to the associated plants. Several *Bacillus* species were found with siderophore production that assisted plant growth promotion [62, 63]. The production of ACC deaminase is an important trait of plant growth-promoting PGPR and endophytic bacteria. ACC deaminase breaks ACC into ammonia and *α*-ketobutyrate. Some studies reported a reduction in the ethylene concentrations when plants were inoculated with ACC deaminase that in turn alleviated the negative effects of stress conditions on plants [64]. The overproduction of ethylene leads to growth reduction in plants under stress conditions [65]. Other studies reported the isolation of *Bacillus* strains with the production of ACC deaminase that improved the growth of inoculated plants [66, 67].

Earlier studies have confirmed the significant role of *Bacillus* species as bioinoculants and biocontrol agents in plant growth promotion [68]. Other than direct effects on plant growth, the rhizospheric and endophytic bacteria may also have an essential role in adaptation of plants to environmental stress conditions. Zhang et al. [69] reported the complete genome sequence of *B*. *halotolerans* ZB201702 that revealed many putative gene clusters involved in defense mechanisms. The isolated strain ZB201702 exhibited drought and salt stress tolerance. The ACC deaminase-producing bacterium *Bacillus licheniformis* HSW-16 and an endophytic halotolerant *Bacillus velezensis* FMH2 were reported to confer salt stress tolerance in wheat and tomato plants, respectively [70, 71]. Both strains protected the plants from growth inhibition by NaCl and increased plant growth. Other studies demonstrated growth promotion in inoculated plants with *Bacillus* strains [72, 73]. Parallel to the above studies, the isolated strain LBG-1-13 in the present study demonstrated growth improvement in Tresor and Bright Diamond under greenhouse and field levels, respectively. Especially, the LBG-1-13-inoculated plants of the Tresor variety showed improved growth performance, particularly that of root growth development. In *in vitro* studies, the strain exhibited enhanced tolerance to salt and drought stresses. Also, the strain LBG-1-13 was found with the activity of ACC deaminase production, which might be responsible for its high tolerance to salt and drought stresses. Therefore, this strain has the potential to be used as a microbial agent under drought and saline conditions due to its property of longer roots. This possibility could be tested in the follow-up study. Some previous studies have demonstrated the important role of *B. halotolerans* in plant growth promotion and alleviation of stress conditions. El-Akhdar et al. [59] characterized two isolated species, *B. halotolerans* and *Lelliottia amnigena*, as PGPR. The strains were further tested for stress tolerance in wheat plants under pot and field experiments, which showed promising results. Recently, Jiménez-Gómez et al. [74] described that *B. halotolerans* SCCPVE07 and *R. laguerreae* PEPV40 strains could be used as efficient bioinoculants for escarole crops. In this study, an in silico genome study of the isolated strains showed the identification of coding regions involved in mechanisms governing plant growth promotion. Both strains conferred plant growth promotion under normal and saline stress conditions. In the light of these studies, the isolated strain LBG-1-13 could further be used to evaluate its potential of conferring lily's and other plants' stress tolerance.

Furthermore, the result of the lily field experiment showed that the strain LBG-1-13 had the function of extending the growth period of the lily compared to uninoculated control plants. For lily production, the plants usually complete the vegetative growth stage and mainly grow above the ground before the flowering stage. While after the flowering stage, it is in the reproductive growth stage in which lily mainly grows the underground bulb part. Therefore, this feature of extending the growth period is very important for lily production, as it has the potential of increasing the yield of Lily. The present study, therefore, generates valuable information towards the application of plant growth-promoting endophytic bacteria as bioinoculants and biocontrol agents.

## 5. Conclusion

The screening of endophytic bacteria capable of antifungal and plant growth promotion traits is important for the overall growth improvement and biocontrol of various lily diseases. We obtained one endophytic bacterial strain, *B. halotolerans* LBG-1-13, from the lily roots which not only antagonized against three kinds of fungal pathogens of lily but also simultaneously had multiple PGP characters including production of IAA, phosphate solubilization, production of siderophores, and ACC-deaminase activity. The strain showed a high potential for salt and drought tolerance. The lily plants inoculated with the isolated bacterial strain showed an improved growth under greenhouse and field conditions. Owing to the antifungal nature and PGP traits, particularly, the ACC deaminase production ability and potential of conferring salt and drought stress tolerance, make this strain a strong candidate as a biocontrol and bioinoculant agent.

## Figures and Tables

**Table 1:** Analysis of plant growth promotion in Tresor variety upon inoculation of the endophytic bacterial strain LBG-1-13.

Treatments	Plant height (cm)	Leaf length (mm)	Leaf width (mm)	Root length (cm)	Root dry weight (g)
CK	45.3 ± 2.2^*a*^	87.5 ± 5.8^*a*^	10.6 ± 1.3^*a*^	19 ± 2.45^*a*^	4.9 ± 0.7^*a*^
LBG-1-13	47.2 ± 2.8^*a*^	94.2 ± 6.7^*a*^	10.9 ± 1.1^*a*^	36.8 ± 2.8^*b*^	11.7 ± 1.7^*b*^

Values are averages ± SD. Values in a column with different superscripts show significant differences based on Student's *t*-test (*p* < 0.05).

## Data Availability

The data used to support the findings of this study are available from the corresponding authors upon request.
